# Measurement Technique Comparison in the Entire Fracture Surface Topography Assessment for Additively Manufactured Materials

**DOI:** 10.3390/ma18061355

**Published:** 2025-03-19

**Authors:** Dawid Zieliński, Aleksandra Mirowska, Przemysław Podulka, Cho-Pei Jiang, Wojciech Macek

**Affiliations:** 1 Faculty of Mechanical Engineering and Ship Technology, Gdańsk University of Technology, 11/12 Gabriela Narutowicza, 80-233 Gdańsk, Poland; dawid.zielinski@pg.edu.pl (D.Z.); aleksandra.laska@pg.edu.pl (A.M.); 2 Advanced Materials Center, Gdańsk University of Technology, 11/12 Gabriela Narutowicza, 80-233 Gdańsk, Poland; 3 Faculty of Mechanical Engineering and Aeronautics, Rzeszow University of Technology, Powstancow Warszawy 12, 35-959 Rzeszów, Poland; p.podulka@prz.edu.pl; 4 Department of Mechanical Engineering, National Taipei University of Technology, Taipei 10608, Taiwan; 5 High-Value Biomaterials Research and Commercialization Center, National Taipei University of Technology, Taipei 10608, Taiwan

**Keywords:** LPBF-printed specimens, measurement techniques, surface metrology, entire fracture surface

## Abstract

This paper focuses on comparing the three microscopic measurement techniques, confocal, focus variation, and point for focus, for the evaluation of entire fracture surface topographies. The measurements were performed using a Sensofar S Neox 3D optical profilometer and the Mitutoyo QV Apex 302 vision measuring system. The test specimens required for measuring were printed through laser powder bed fusion (LPBF) technology using two materials: Stainless Steel 316L and Inconel 718. The printing was performed with a printing power of 200 W, scanning speed of 800 mm/s, and layer thickness of 30 µm or 50 µm. The measurement differences were analyzed on the basis of void volume *(Vv)*, fractal dimension (*Df)*, and texture isotropy parameters, as well as a general view of the surface topography. The obtained results did not show a comprehensible difference between the applied measurement techniques for particular specimens. Thus, both measurement devices and three measurement techniques can be used to precisely measure the dimensions of LPBF-processed specimens with the *entire fracture surface* method.

## 1. Introduction

Surface roughness is one of the most basic output values of any manufacturing process, including 3D printing technology. The condition of the surface structure has a significant impact on the structural and functional properties of the mechanical components. Thus, it is of prime importance to use appropriate methods to evaluate and analyze the surface topography of 3D-printed components. In general, measurement methods can be divided into mechanical and electromagnetic contact and non-contact methods, which enable measurements in 2D and 3D systems [1,2]. Over the past few decades, a diversity of surface topography measuring instruments [3] have been expanded and made commercially available due to their efficiency and adaptability to the form and texture measurement of complex surfaces.

Stylus instruments are basic metrological devices that are mainly used to measure characteristics such as surface roughness and waviness [4]. Measurement data can be collected along a measurement section (2D system) or a specific area (3D system—surface topography). Unfortunately, measuring roughness with the use of a contact stylus tracing method is intrusive. Thus, it cannot be used to evaluate the surface of porous parts with high roughness, such as mechanical components printed by laser powder bed fusion methods [5,6]. Therefore, there is a growing demand for non-contact and online methods for measuring these types of surfaces [7]. Currently, 3D optical profilometers are becoming more and more important in enabling the realization of accurate and repeatable measurements using various measurement techniques, such as confocal [8], interferometric [9], and focus variation [10]. For example, the authors of [11] used different optical devices and techniques based on confocal and focus variation techniques to measure a Ti6Al4V titanium alloy surface after finish turning under dry machining conditions. The obtained results indicated a clear measurement difference between the devices used, which reached up to ~30%. In another study, Podulka et al. [12] used a focus variation microscope and confocal measurement techniques to evaluate entire bending-fatigued fracture surface topographies for specimens obtained by explosive welding. Differences in the obtained measurement results related to the occurrence of errors in the form of outliers and noise were demonstrated.

Additionally, non-contact instruments are improved by the occurrence of measurement noise [13,14]. Areal surface topography measurement results from twelve optical surface topography instruments, including focus variation, confocal microscopes, and coherence scanning interferometers, were compared, and good-practice guidance for regular users for quantifying, specifying, and interpreting measurement noise was defined [15]. Further, international standards propose repetition of the measurement process and averaging the obtained results [16]. Some alternative procedures have been proposed using surface topography analysis functions in the correct order with sophisticated application conditions [17].

Laser powder bed fusion (LPBF) is gaining popularity due to its production capabilities and reduced power consumption. In particular, it has emerged as an alternative method for producing metal parts with new developments in materials [18,19]. The technology currently faces different metallurgical irregularities such as porosity, lack of fusion, residual stresses, micro-cracks, or high surface roughness, which greatly affect the mechanical performance of the printed parts [20,21,22]. The LPBF Inconel 718 alloy’s surface topography was studied to determine the occurrence of measurement noise when using a non-contact white light interferometer [23].

Considering the need to verify the universality of the *entire fracture surface* method as well as other procedures for quantifying and interpreting fracture surface topography, the authors decided to conduct measurements using a variety of approaches. In this study, two different optical systems, a Sensofar S Neox 3D (Sensofar, Terrassa, Spain) optical profilometer and the Mitutoyo QV Apex 302 (Mitutoyo, Kawasaki, Japan) vision measuring system, were used to measure the entire fracture surface metrology for 3D-printed (LPBF) specimens using Stainless Steel 316L and Inconel 718.

## 2. Materials and Methods

### 2.1. Characteristics of Test Specimens and Surface Measurement Devices

**Figure 1:**
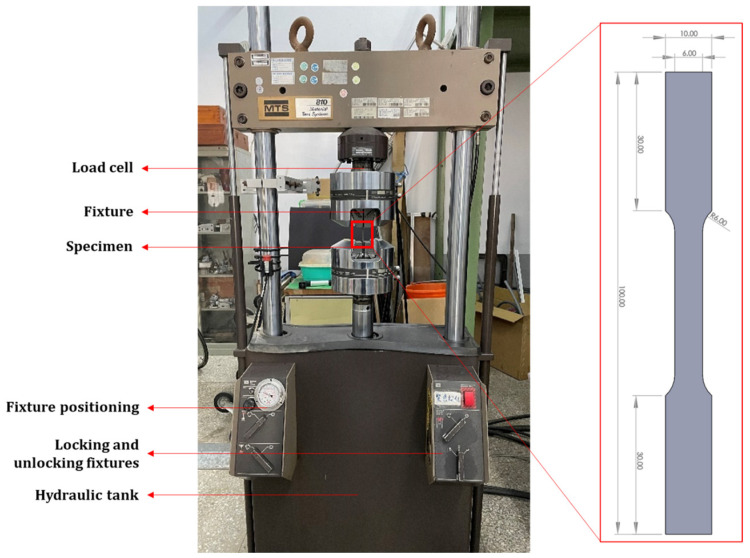
Tensile testing of the specimen fabricated using the LPBF process as per ASTM E8 standards.

The test specimens were fabricated using the Tongtai AMP-160 (Tongtai Co., Kaohsiung, Taiwan) laser powder bed fusion 3D printer. The process parameters used to fabricate the specimens are listed in Table 1 and were fed into the slicer software Materialise Magics 23.1 (Leuven, Belgium). The tensile specimen is present in Figure 1, and the printing direction of the specimen is perpendicular to the tensile direction, which allows for higher tensile strength compared to the parallel direction. Once the specimens are printed, they need to be separated from the build platform. This is carried out using Wire Electrical Discharge Machining (WEDM). Post-processing is carried out with utter care to avoid electrical discharges damaging the specimens.

Utilizing these process parameters, the tensile specimens were fabricated following the ASTM E8 standards, as shown in Figure 1. The tensile testing was performed using an MTS-810 universal testing machine (MTS Systems Corporation, Eden Prairie, MN, USA). The tensile testing was performed using a 100 kN load cell and a strain rate of 0.05 mm/s. The constant surge in the load resulted in a tensile deformation of the specimen until failure. As soon as the specimen broke (i.e., failed), the loading stopped, thereby ending the testing.

**Figure 2:**
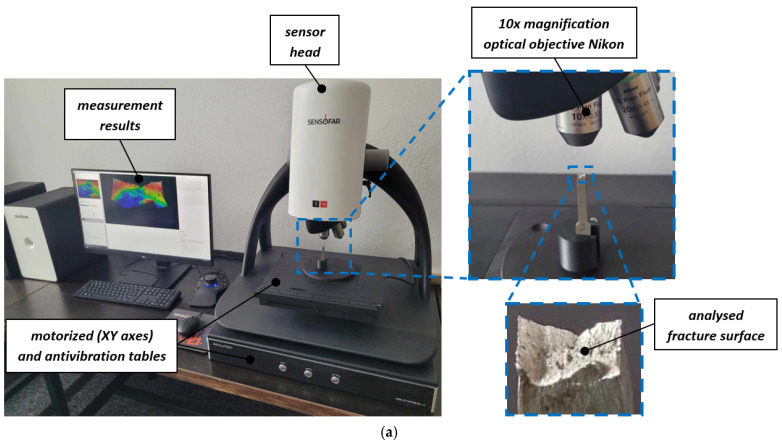

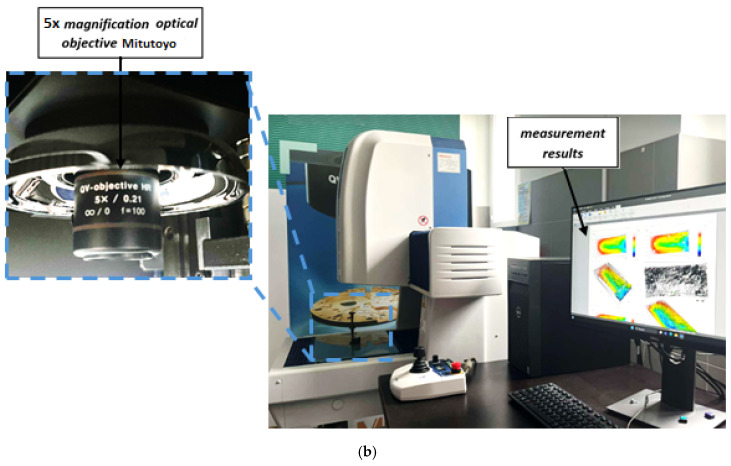
Measurement devices used to evaluate surface topography: (**a**) Sensofar S Neox 3D optical profilometer; (**b**) Mitutoyo QV Apex 302 vision measuring system.

Surface topography measurements for additively manufactured materials were performed using a Sensofar S Neox 3D optical profilometer based on confocal and focus variation microscope techniques and the Mitutoyo QV Apex 302 vision measuring system (Figure 2). Sensofar S Neox, representing 3D optical profilers based on confocal microscopy and interferometry, is used for the measurement and characterization of micro- and nano-scale features on natural or manufactured surfaces. Three-dimensional workpiece measurements can be performed with Mitutoyo QV Apex 302, which belongs to 3D Coordinate Measurement Machine (CMM)-type devices. These enable the 3D measurement of workpieces such as press-molded products, plastic-molded products, and cut products, which until recently could not be measured with image processing alone. The crucial measurement conditions for each device are given in Table 2. In each case, the measurement field included the entire fracture for the analyzed specimen. Measurements by confocal and focus variation techniques were carried out with a Nikon–EPI 10× magnification objective and in SensoSCAN S neox 7.7 software. Measurements were also performed with the use of the Mitutoyo QV Apex 302 vision measuring system under 5× magnification with a 1.24 × 0.93 mm^2^ field of view, and were stitched together to map the entire fracture area, with a total resolution scale of 0.1 μm. Collected Mitutoyo (*.Q3D) data files were transferred to the surface texture analysis software MountainsMap 8.0 and resampled into height maps at a resolution automatically set by the software. The ISO 25178 surface topography parameters were calculated and analyzed. No additional filters were used.

### 2.2. Measurement Procedure

**Figure 3:**
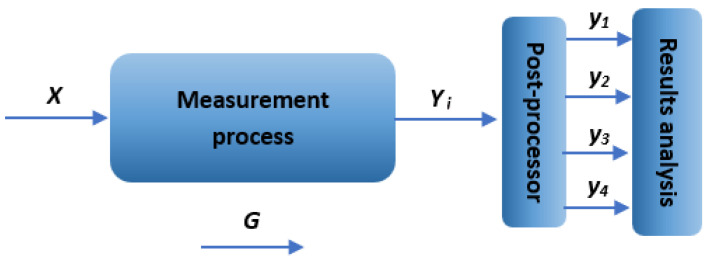
Scheme of the measurement process using dedicated devices.

Measurements using different devices were carried out according to the procedure presented in Figure 3. The obtained raw measurement data were then analyzed with the use of the specialized software MountainsMap premium 9.1 as a post-processor. The final measurement results concerned void volume (*Vv)*, *Df* (enclosing box method), and texture isotropy, as well as a general view of the surface topography. In this study, surface topography measurements were conducted for specimens made of two materials: Stainless Steel 316L and Inconel 718. In total, 5 entire fracture surfaces were included in the analysis, including 3 from Stainless Steel 316L and 2 from Inconel 718. Measurements of each surface were performed once using the measurement systems and techniques analyzed—Table 2.

#### Characteristics of Factors

**X—input factors:** *measurement techniques and methods—Sensofar S Neox 3D optical profilometer (confocal and focus variation microscope techniques), Mitutoyo QV Apex 302 vision measuring system.*
**Y—output factors (analyzed values):** *y_1_—void volume Vv [mm³/mm²]; y_2_—Df* enclosing boxes *[-]; y_3_—texture isotropy [%]; y_4_—general view of the surface topography [isometric images].*
**G—constant values:** *parameters and measurement conditions for each device,* etc.

### 2.3. Measurement Data Analysis Procedure

**Figure 4:**
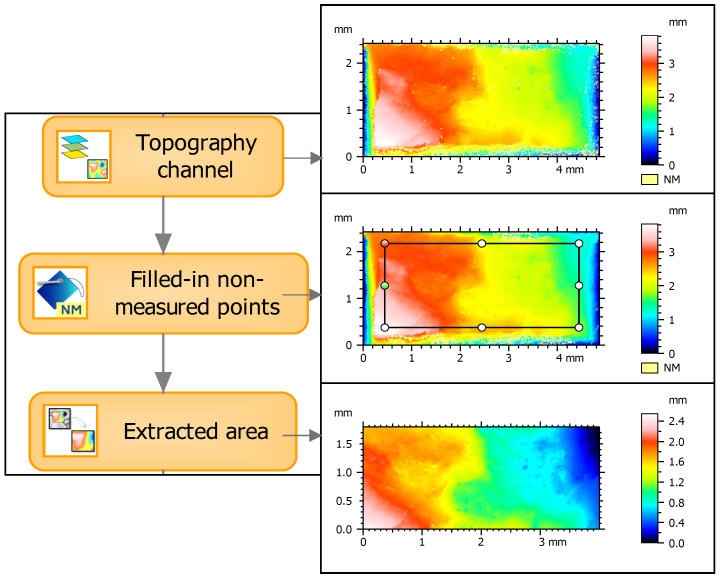
Schematic view of algorithm and original, filled-in, and final extracted fracture surface area.

The obtained measurement data from both devices were transferred to MontainsMap Premium 9.1 software. The same data analysis procedure was used for each specimen and measurement technique. The whole surface was reduced to eliminate the regions associated with geometric discontinuities or missing points as well as to obtain uniform dimensions for all specimens, according to the entire fracture surface method proposed by Macek [24]. An example of the measurement data processing is shown in Figure 4. First, the channel topography was separated. Next, the non-measured points were eliminated using the smooth shape calculated from the neighbor method. Finally, the surface was limited to a rectangle with dimensions of 1.8 × 4.0 mm^2^.

### 2.4. Scanning Electron Microscope Observations

The fracture surfaces of the steel and Inconel specimens were observed with a high-resolution scanning electron microscope (SEM JEOL JSM-7800 F, JEOL Ltd., Tokyo, Japan) with the use of an LED detector operating at an acceleration voltage of 5.0 kV.

## 3. Results

**Figure 5:**
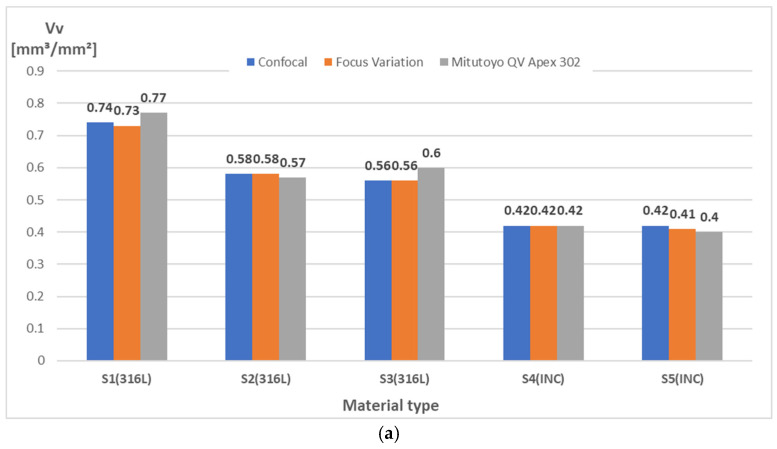

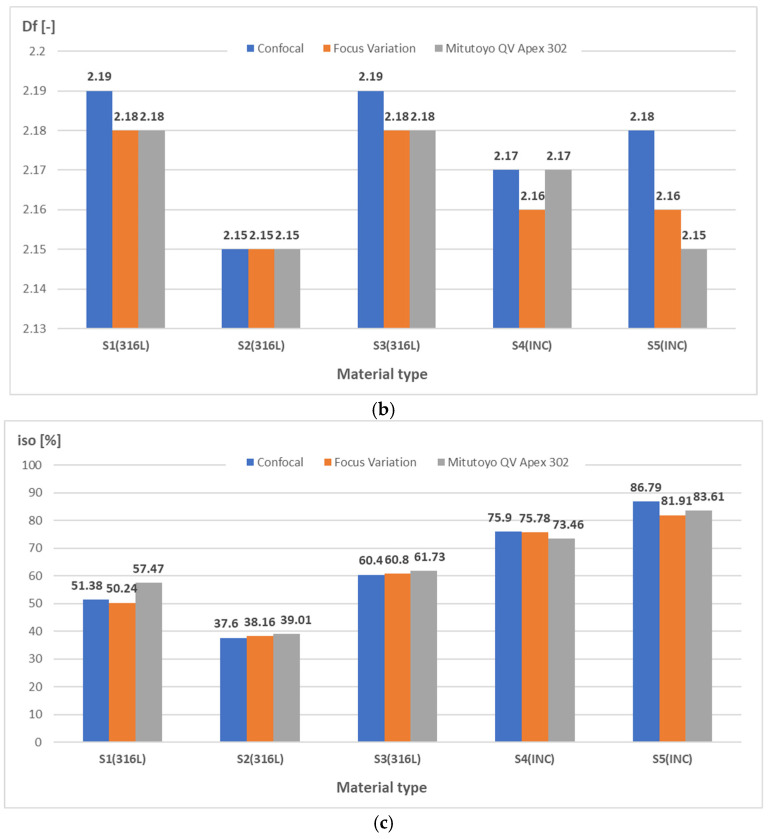
Comparison of measurement results for different materials and measurement techniques: (**a**) *Vv*—void volume; (**b**) *Df*—enclosing boxes; (**c**) *iso*—texture isotropy.

**Figure 6:**
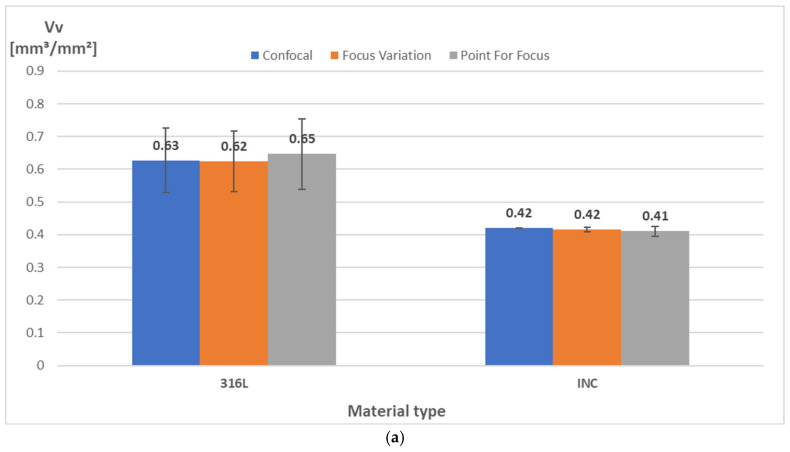

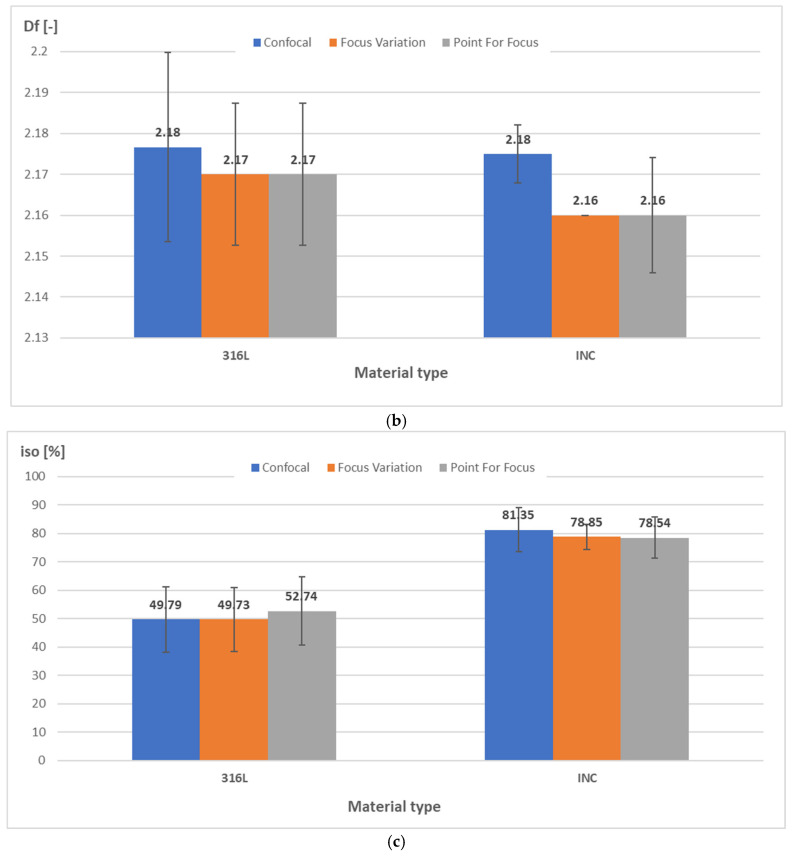
Average values of analyzed measurement results for different materials and measurement techniques: (**a**) *Vv*—void volume; (**b**) *Df*—enclosing boxes; (**c**) *iso*—texture isotropy; error bars show ± the standard deviation.

**Figure 7:**
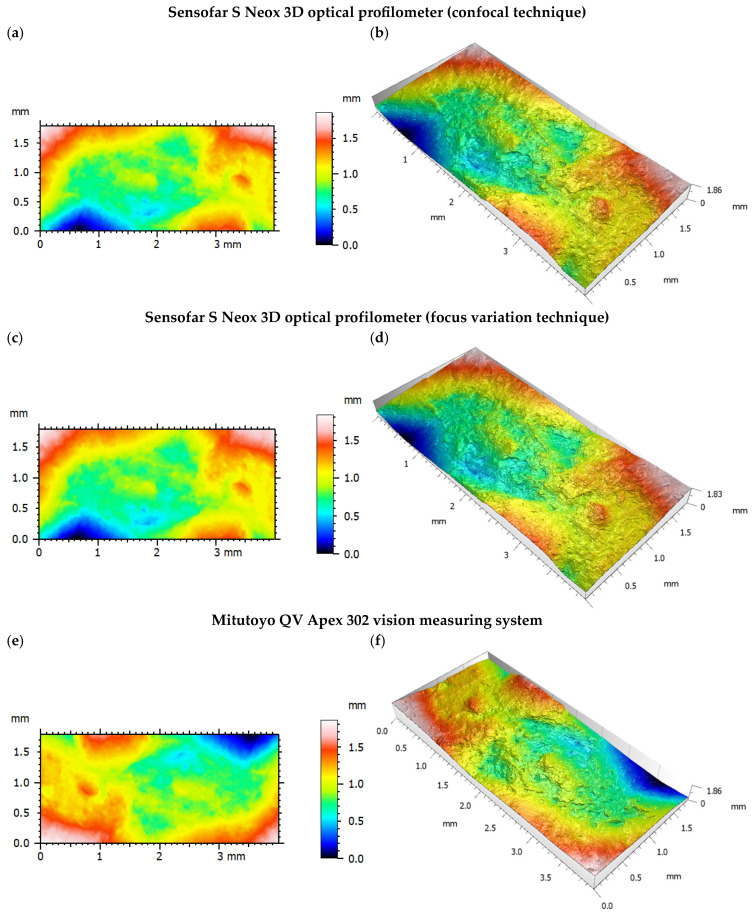
Three-dimensional evaluation of the entire fracture surface topography—specimen S4(INC) measured by the Sensofar S Neox 3D optical profilometer and Mitutoyo QV Apex 302 vision measuring system: (**a**,**c**,**e**) pseudo-color views of the surfaces; (**b**,**d**,**f**) isometric views of the surfaces.

The obtained measurement results for each of the devices and types of materials tested are included in Table 3, while their comparison is presented in Figure 5 and Figure 6. Moreover, Figure 7 shows isometric views and 3D images of the entire fracture surface topography for specimen S4(INC) measured by the Sensofar S Neox 3D optical profilometer with confocal and focus variation methods, as well as the Mitutoyo QV Apex 302 vision measuring system. For the same surfaces of test specimens, very similar results of the analyzed outputs were obtained using different measurement techniques.

The void volume *Vv* is the volume of space bounded by the surface texture from a plane at a height corresponding to a chosen value to the lowest valley. *Vv* indicates a measure of the void volume provided by the surface between various heights. To use volume parameters, the areal material ratio values that divide the reduced peaks and reduced valleys from the core surface must be specified. By default, 10% and 80% are used.

The fractal dimension *Df* allows the use of fractional geometric dimensions, for instance for surfaces of dimensions between 2 and 3. A surface with a fractal dimension of 2.15 looks thus less complex than one with a dimension of 2.19. It is falsely said to be ’nearer’ to a plane (2D) than to a volume (3D). The actual surface area is of course the same in both measurement cases.

Surface topography analysis complements a texture isotropy study, which involves calculating the isotropy parameters with respect to a threshold, assumed equal to 0.20. These values allowed us to quantify the central zones corresponding to the portion of the peaks that remained after thresholding. Isotropy in a surface indicates that the surface maintains the same structure properties in all directions.

It can be observed that higher *Vv* parameters were obtained for Stainless Steel 316L specimens, which show fractures of a more brittle nature. Plastic fractures, in general, show a smoother surface structure, which is reflected in the lower *Vv* values, as can be noted for Inconel 718 specimens. Brittle fractures are usually characterized by surfaces with large height differences, as reflected by high values of *Vv*. The obtained values of the *Df* parameter indicate slightly higher values for specimens made of 316L Stainless Steel. Upon analyzing the surface isotropy values for the two types of specimens tested, a notable difference emerges in the results (see Figure 6). The 316L Stainless Steel specimens yielded a value of 53.7%, whereas the Inconel 718 specimens demonstrated a higher average of 77.8%.

**Figure 8:**
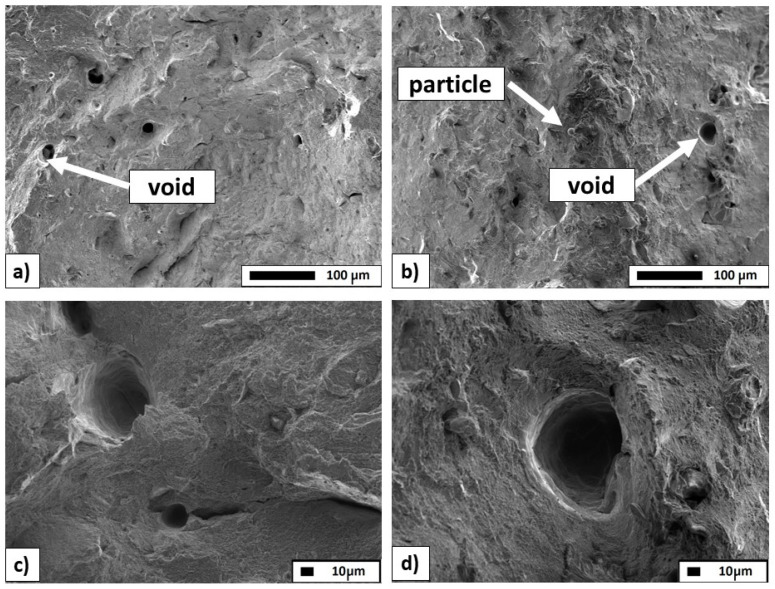
Scanning electron microscopy fracture images of 316 L (**a**,**c**) and Inconel 718 specimens (**b**,**d**).

To analyze the nature of the fractures, SEM imaging was also performed. Figure 8 shows the images at different magnifications for both materials. In the 316L Stainless Steel specimen (Figure 8a,c), both longitudinal and transverse cracks are present, with a ductile fracture. Small porosities, which may weaken the specimen, were noted, but no unmelted powder grains were found. In the Inconel 718 specimen (Figure 8b,d), transverse cracks dominate, and the fracture is also ductile, with significant porosity and unmelted powder grains observed.

## 4. Discussion

A systematic investigation of fracture surface topography is essential for identifying the dominant failure and damage mechanisms in service. Advanced surface measurement techniques have been developed to inspect the damage mechanisms responsible for structural failures. The analysis demonstrated that there is little difference between the results acquired with the optical technique in order to detect the surface topography, which can be used successfully in quantitative fractography, especially in the *entire fracture surface* method. Both measurement systems have been successfully used to evaluate the surfaces of AM-fabricated specimens, showing the great potential of non-contact methods in the analysis of irregularly shaped and highly rough surfaces. This was confirmed by the repeatable and similar values of the analyzed measurement results given in Figure 5, Figure 6 and Figure 7. Simultaneously, considering the models and measurement capabilities of the analyzed systems, there was no clear effect of the measurement technique and parameters used on the analyzed quantities, as well as the shape of the surface topography [25]. For each device, measurements were performed under laboratory conditions, mainly to eliminate vibrations that could affect the quality of the results obtained [26]. The modern industrial sector requires the implementation of rapid, repeatable, and accurate measurements. Thus, it is necessary to use appropriate measuring equipment, especially for AM parts characterized by high roughness and irregular structures. The results obtained in this study confirmed the possibility of using the two analyzed measurement systems in industrial practice to accurately assess the surface of AM parts. In addition to performing measurements with the appropriate equipment and set of parameters, proper analysis of the raw measurement data is also an important issue. The *entire fracture surface* method used in this study allowed for a relatively quick and accurate evaluation of the surface of specimens made of metal powders. The fracture surface topography parameters collected from both apparatuses of the same specimens led to comparable results, which reinforces the applicability of the proposed approach. The *entire fracture surface* method opens perspectives in post-failure characterization, allowing for correlations between the reasons for damage and fracture surface topography characteristics [27]. The consistent surface topography measurements across all the presented devices and techniques suggest that the *entire fracture surface* method effectively assesses the surface structure. The methodology presented in this paper can be directly adopted by the industry, provided that there is an optical profiler, and can be particularly useful to trace back the failure origin as well as to provide important clues in forensic engineering.

## 5. Conclusions

The most important conclusions are as follows:

- The specimens were successfully fabricated using the following laser process parameters: printing power of 200 W, scanning speed of 800 mm/s, hatch spacing of 105 µm, and layer thickness of 50 µm. The specimens were successfully post-processed with the WEDM process.
- SEM observations confirm that both materials exhibit ductile fracture modes. In the Inconel 718 specimens, additional porosities and unmelted powder grains were identified, a result of the additive manufacturing process.
- Optical systems allow for the accurate evaluation of irregularly shaped surfaces, such as those analyzed in this paper and the entire fracture surfaces of AM-fabricated materials.
- Obtaining similar results of surface topography measurements for each of the presented devices and measurement techniques indicates a proper assessment of the structure of the entire fracture surface metrology; both measurement devices can therefore be used to measure the topography of this type of surface for additively manufactured materials.
- Both measurement systems and the demonstrated method of analyzing measurement data can be used in an industrial environment to accurately assess irregularly shaped surfaces, such as AM parts.
- Another key point to note is that the *entire fracture surface* method holds significant promise for analyzing failures in 3D-printed metals functioning under both quasi-static and fatigue conditions. Furthermore, it may greatly assist in understanding the failure mechanisms resulting from various loads.
- Future research directions include testing and detailed analysis of a wider range of AM-fabricated materials, including advanced and hybrid materials. Based on the collected measurement data, it will be possible to further improve current measurement techniques and develop appropriate methods for their analysis.

## Figures and Tables

**Table 1:** Three-dimensional printing conditions for test specimens.

**Machine and Software**	
3D printer	Tongtai AMP-160
Software (slicer)	Materialise Magics 23.1
Method and materials	
3D printing method	LPBF
Materials	Stainless Steel 316L, Inconel 718
Process parameters	
Laser power	200 W
Scanning speed	800 mm/s
Hatch space	105 µm
Layer thickness	50 µm
Post-processing	
Wire Electrical Discharge Machining (WEDM)	

**Table 2:** The crucial measurement conditions for the devices used.

**Parameter**	**Measurement System**	
	**Sensofar S Neox 3D**	**Mitutoyo QV Apex 302**
Light source	LED	LED strobe light
Pixel size	1.38 µm	
Optical resolution	0.46 µm	0.10 µm
Measurement software	SensoSCAN S neox 7.7	FORMTRACEPAK V6
Magnification	10	5
Measurement technique	Confocal, focus variation	Point for focus

**Table 3:** Set of measurement data for analyzed values: *Vv*—void volume; *Df*—enclosing boxes; *iso*—texture isotropy.

**Material Type**	**Measurement Technique**		
	**Confocal** **(Sensofar S Neox 3D)**	**Focus Variation (Sensofar S Neox 3D)**	**Point For Focus** **(Mitutoyo** **QV Apex 302)**
**Analyzed value: *Vv*—void volume [mm³/mm²]**			
S1(316L)	0.74	0.73	0.77
S2(316L)	0.58	0.58	0.57
S3(316L)	0.56	0.56	0.6
S4(INC)	0.42	0.42	0.42
S5(INC)	0.42	0.41	0.4
**Analyzed value: *Df*—enclosing boxes [-]**			
S1(316L)	2.19	2.18	2.18
S2(316L)	2.15	2.15	2.15
S3(316L)	2.19	2.18	2.18
S4(INC)	2.17	2.16	2.17
S5(INC)	2.18	2.16	2.15
**Analyzed value: *iso*—texture isotropy [%]**			
S1(316L)	51.38	50.24	57.47
S2(316L)	37.6	38.16	39.01
S3(316L)	60.4	60.8	61.73
S4(INC)	75.9	75.78	73.46
S5(INC)	86.79	81.91	83.61

## Data Availability

The original contributions presented in this study are included in the article. Further inquiries can be directed to the corresponding authors.
